# The effect of anti-nausea wristbands and STW-5 in children suffering from dyspepsia – a randomized double-blind placebo-controlled pilot-study

**DOI:** 10.1055/a-2652-1298

**Published:** 2025-08-21

**Authors:** Corinne Légeret, Sebastian Ludyga, Muriel D’Aujourdhui, Selina Schneider, Sarah Hagmann, Annemarie Acket, Henrik Köhler, Raoul Furlano

**Affiliations:** 1 Pediatric Gastroenterology, University Childrens Hospital Basel, Basel, Switzerland; 2 Department of Sports, Movement and Health, University of Basel, Basel, Switzerland; 3 Medical Faculty, University of Basel, Basel, Switzerland; 4 Children’s Hospital, Kantonsspital Aarau, Aarau, Switzerland

**Keywords:** children, functional dyspepsia, anti-nausea bands, Placebo, Kinder, funktionelle Dyspepsie, anti-Nausea Bänder, Placebo

## Abstract

**Background:**

Functional dyspepsia (FD) is common in children and refers to discomfort in the epigastric/duodenal region, which cannot be explained by structural or biochemical abnormalities. Since the pathophysiology is still not fully understood, no guidelines in terms of treatment do exist.

**Aim:**

The aim of this study was to compare the therapeutical effect of plant extracts (STW-5) and acupressure in children with functional dyspepsia.

**Methods:**

This is a randomized, double-blind placebo-controlled trial. Before and after the allocated intervention of four weeks, participants had to fill in a cross-cultural validated questionnaire consisting of 52 questions to assess quality of life, as well as the extent of their discomfort (using a standardized visual analog scale (VAS)).

**Results:**

33 patients were included in the study: 12 male and 21 female with an average age of 12.09 years (range: 7–17 years). Symptoms improved after four weeks of intervention in most treatment-groups: By 68% in children, who received STW-5, by 40% in the wristband (WB)- group and by 35% in the wrist-band-Placebo-group (WBPL). Patients receiving STW-5- Placebo showed even an increase of symptoms.

**Discussion:**

This double-blind, placebo-controlled pilot study showed, that STW-5 and/or anti-nausea wristbands are a cheap and effective option to treat children and teenagers suffering from functional dyspepsia.

## Zusammenfassung

**Hintergrund:**

Funktionelle Dyspepsie (FD) ist bei Kindern häufig und bezieht sich auf Beschwerden in der epigastrischen/duodenalen Region, die nicht durch strukturelle oder biochemische Anomalien erklärt werden können. Da die Pathophysiologie noch immer nicht vollständig geklärt ist, gibt es keine Leitlinien für deren Behandlung.

**Ziel:**

Das Ziel dieser Studie war es, die therapeutische Wirkung von Pflanzenextrakten (STW-5) und Akupressur bei Kindern mit funktioneller Dyspepsie zu vergleichen.

**Methoden:**

Es handelt sich um eine randomisierte, doppelblinde, Placebo-kontrollierte Studie. Vor und nach der jeweiligen vierwöchigen Intervention mussten die Teilnehmer einen kulturübergreifenden, validierten Fragebogen mit 52 Fragen ausfüllen, um die Lebensqualität sowie das Ausmaß ihrer Beschwerden (anhand einer standardisierten visuellen Analogskala (VAS)) zu bewerten. Ergebnisse: 33 Patienten wurden in die Studie eingeschlossen: 12 Männer und 21 Frauen mit einem Durchschnittsalter von 12,09 Jahren Range: 7–17 Jahre). Die Symptome verbesserten sich in den meisten Behandlungsgruppen nach der vierwöchiger Behandlung: Um 68% bei Kindern, die STW-5 erhielten, um 40% in der Armband (WB)-Gruppe und um 35% in der Armband-Placebo-Gruppe (WBPL). Patienten, die STW-5-Placebo erhielten, zeigten sogar eine Zunahme der Symptome.

**Diskussion:**

Diese doppelblinde, Placebo-kontrollierte Pilotstudie hat gezeigt, dass STW-5 und/oder Anti-Nausea-Armbänder eine kostengünstige und wirksame Option zur Behandlung von Kindern und Jugendlichen sind, die an funktioneller Dyspepsie leiden.

## What is known

▪many children suffer from functional epigastric discomfort ▪ the pathophysiology is complex, therefore no standard guidelines for the treatment exist

## What is new

▪STW-5 and anti-nausea wristbands are a cheap and effective options to treat children suffering from functional dyspepsia ▪a reduction of gastrointestinal symptoms did not correlate with an improvement of cognitive performance

## Background

Functional gastrointestinal disorders (FGIDs) are very common in children of all ages. It is an umbrella term for discomfort in any part of the gastrointestinal tract, which cannot be explained by structural or biochemical abnormalities 1 . FGIDs are diagnosed according to the symptom-based Rome criteria, which have been revised in 2016 (Rome IV criteria, 2 ) and rely on the medical history and physical examination. Dyspepsia refers to the Greek words ‘dys’ and ‘peptos’ and is translated with ‘hard to digest’. Functional dyspepsia (FD) is a sub-group of FGIDs, it is a clinical syndrome comprising chronic symptoms arising from the gastroduodenal region. According to the Rome criteria, the prototypical symptoms are postprandial fullness, early satiation, epigastric pain or burning not associated with defecation. The diagnosis of FD can be made in children and teenagers, once one of those symptoms occur a minimum of four times a month for at least two months and after appropriate evaluation, to ensure that the symptoms cannot be explained by another medical condition 2 . The pathophysiology is still not fully understood, but it seems to rely on a combination of visceral hypersensitivity, motility disturbances, alteration in gastrointestinal microbiota, mucosal and immune function and central nervous processing 3 .

Treatment of FD is challenging and based on empiric strategies due to the lack of published treatment trials on chronic nausea, especially in children. Studies suggest that hypnotherapy targets the aetiologic biopsychosocial factors and therefore modifies visceral hypersensitivity 4 .

Herbal medications have multiple mechanisms of action in virtue of diverse components potentially regulating various, complex aetiologies simultaneously and comprehensively improve symptoms of FGIDs. The herbal combination STW-5 (Iberogast) was shown to relax muscle activity in the fundus area of the stomach but to enhance the contractions in the antrum area in an animal study 5 . In a study with healthy adults, administration of STW-5 led to a significant increase in proximal gastric volume measured with a gastric barostat, whilst the antral pressure wave increased 6 . STW-5 is a liquid preparation including fresh plant extracts from eight dried herbs and is sold across Europe as over-the-counter medication. Overall, it regulates motility by acting on the gastrointestinal smooth muscles in an area-specific manner, stimulates gastric secretion and exerts spasmolytic, choleretic, anti-inflammatory and carminative effects 7 8 . Several studies proved its efficacy in adults suffering from FD 9 10 , but no data for children is available.

Another – on first sight unusual – approach to overcome nausea is the use of acupuncture (needling), acustimulation (electrical stimulation) or constant pressure (acupressure) to the P6 acupuncture point (located on the side of the wrist), which is recognized as a target pint in Chinese and acupuncture medicine for reducing nausea and vomiting. It seems, that the effect of this method is an electrical stimulus of low frequency on sensory receptors in the skin, which are activators of A delta and A beta fibers. These fibers synapse in the posterior horn and this might result in release of endorphin in the hypothalamus 11 . The increase in beta-endorphin concentration in human CSF after acupuncture stimulus has been described 12 .

The aim of this study is to compare the therapeutical effect of plant extracts and acupressure in children with functional dyspepsia and perform a standardized test of cognitive psychology.

## Methods

This is a randomized controlled double-blind trial of paediatric patients with FD. Children between the age of 6 and 18 years, who complained about gastric symptoms (pain, nausea, fullness) and had a normal full blood count, a normal thyroid function, a negative coeliac screening (normal t-transglutaminase antibodies, normal total IgA) and most importantly normal macro- and microscopic findings on oesophago-gastro-duodenoscopy (all had biopsies taken from duodenum, stomach and oesophagus) were informed about the study between September 2016 and September 2020. Exclusion criteria were gastrointestinal infection less than two weeks ago, an underlying gastrointestinal disease, use of drugs two weeks prior to starting the study. After signing the informed consent by the participant and/or legal representative respectively, patients were asked to fill in the Kidscreen-52, a cross-cultural validated questionnaire consisting of 52 questions to assess the health-related quality of life 13 , as well as the extent of their discomfort (using a standardized visual analog scale (VAS), points 1–10). In addition, participants had to take a quick test on the computer: Eriksen flanker task 14 , a validated task used to study cognitive processing, attention and control processes (e. g. interference, activation and inhibition). Patients themselves drew a lot, where the type of treatment (Iberogast/wrist band) was determined, of course neither the patient nor the study nurse knew whether an original product or the placebo equivalent was given. Patients were randomized allocated to four different treatment-groups

1. Children get treated with STW-5 (Iberogast (IB)), dosage as per recommendation of the producer (Children<12 years: 3x 20 drops a day; Children<12 years: 3x15 drops a day): They received the original STW-5 in a neutral bottle without primary package and label

2. Children get treated with an Iberogast-Placebo (IBPL), same dosage as original STW-5: They received the same neutral bottle, containing Placebo. To get the same acerbity as the original product, quinine and brown food coloring for the appearance was used. This was also prepared and blinded by the Hospital Pharmacy.

3. Children receive a wristband (WB) with the effect of an acupressure (psiBands): They received an official acupressure wrist band, that must be worn over the whole time (the correct area is in between the two tendons on the underside of the wrist- two finger widths from first crease in wrist) without the original packaging

4. Children receive a Placebo wristband ((WBPL), same wristband without an effect of acupressure), which must be worn all the time (the correct area is in between the two tendons on the underside of the wrist- two finger widths from first crease in wrist) without the original packaging.

**Fig. 1:**
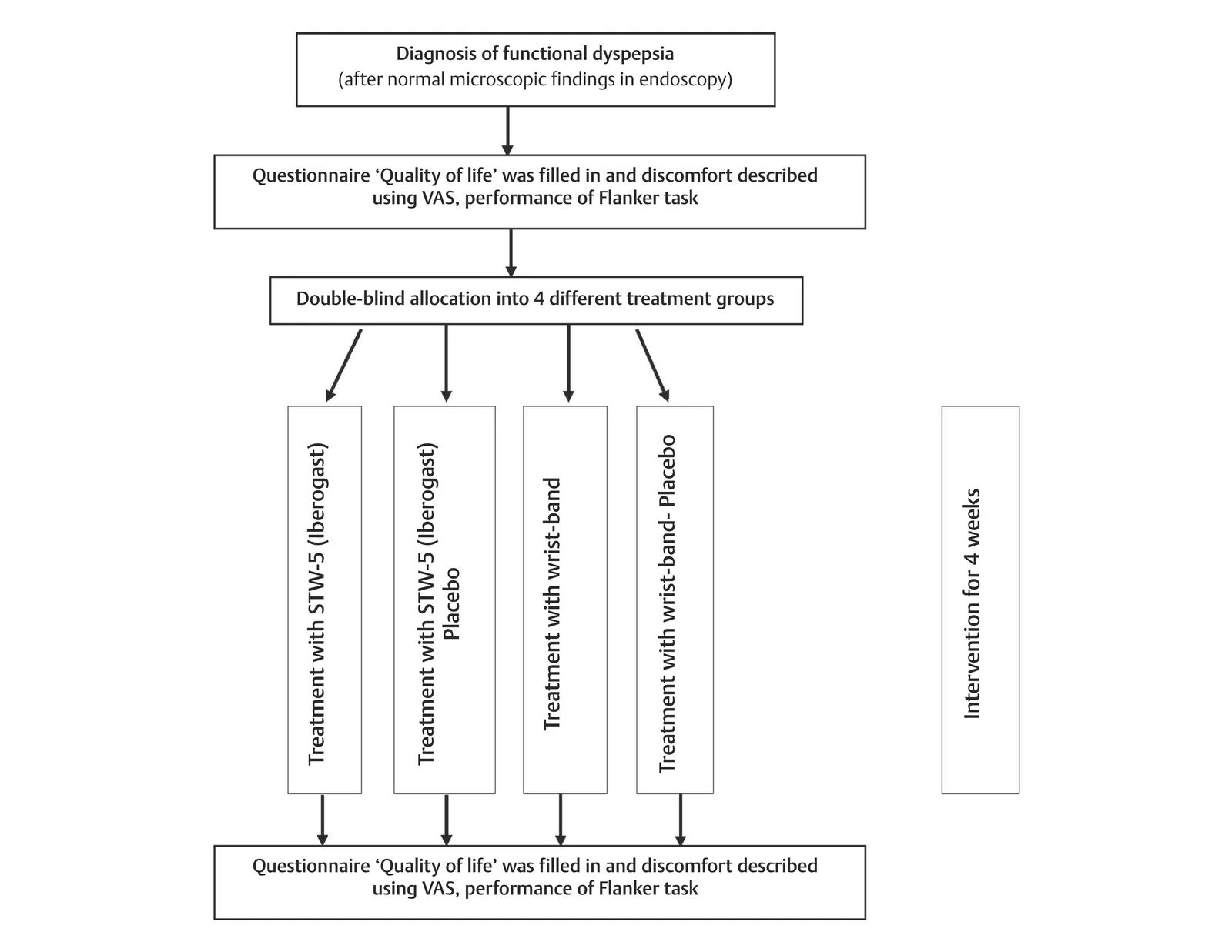
Study method.

Participants had to take the allocated medication or wear the wristbands for a total of four weeks and keep a diary about the daily complaints. At the end of the four weeks, participants had to fill out the same questionnaire concerning quality of life and extent of medical condition, and they were asked to re-do the flanker task. Fig. 1 displays the course of the study.

### Statistical analysis

Statistical analysis was performed with SPSS 22.0 for Windows. As the visual analog scale possesses interval and ratio properties, it was treated as numerical data. One-way ANOVA was used to compare baseline values between the four groups. Subsequently, 4 groups by 2 (time: baseline, post-intervention) ANOVA with repeated measures on the second factor was employed to analyse the effect of therapy on functional nausea. Main effects and interactions were reported. When significant F-ratios were obtained, Bonferroni was used as post hoc test for individual comparison. For all statistical comparisons, an alpha level of p≤0.05 was considered significant. Eta-squared (eta ^2^ ) was measured as effect size and interpreted after Cohen 15 as following: > 0.01 (small), > 0.06 (medium) and >0.14 (large).

### Ethical statement

The present study was approved by the ethical committee. Furthermore, the study was conducted in accordance to the ethical principles laid down in the Declaration of Helsinki and its later amendments.

## Results

From 273 children suffering from nausea with normal macroscopic and histological findings in an upper endoscopy, 60 patients agreed to participate in the study, of which 27 terminated the participation due to fading symptoms whilst the trial. Therefore, a total of 33 patients were included in the study: 12 male and 21 female with an average age of 12.09 years (7–17 years), an average weight of 47.2 kg (range: 22–105 kg) and an average length of 151 cm (range: 120–179 cm). For further patient characteristics and allocation to the different treatment groups, we refer to Table 1 .

**Table:** **Table 1** Patient’s characteristics

	Male				Female				In total (female and male)			
	n	Mean age (years)	Mean weight (kg)	Mean height (cm)	n	Mean age (years)	Mean weight (kg)	Mean height (cm)	n	age in years (range)	weight in kg (range)	Height in cm (range)
IBPL	2	13.5	48.2	155.5	4	14	67	168	6	13.8 (12–16.5)	60.7 (34–90)	163.8 (153–179)
IB	1	13.5	42.5	168	6	12.4	47.4	149.8	7	12.6 (8.3–17.8)	46.7 (18.8–105)	152.4 (122–179)
WB	6	9.9	31.8	134.2	9	11.4	40.8	147.7	15	10.8 (7.5–17)	37.3 (20–65)	142.3 (117–170)
WBPL	3	9.8	38.8	136.6	2	11.25	35.5	142	5	10.4 (6.5–12.5)	37.5 (26–59.5)	138.8 (122–155)

IBPL: Iberogast-Placebo; IB: Iberogast; WB: Wrist band; WBPL: Wrist band Placebo

**Fig. 2:**
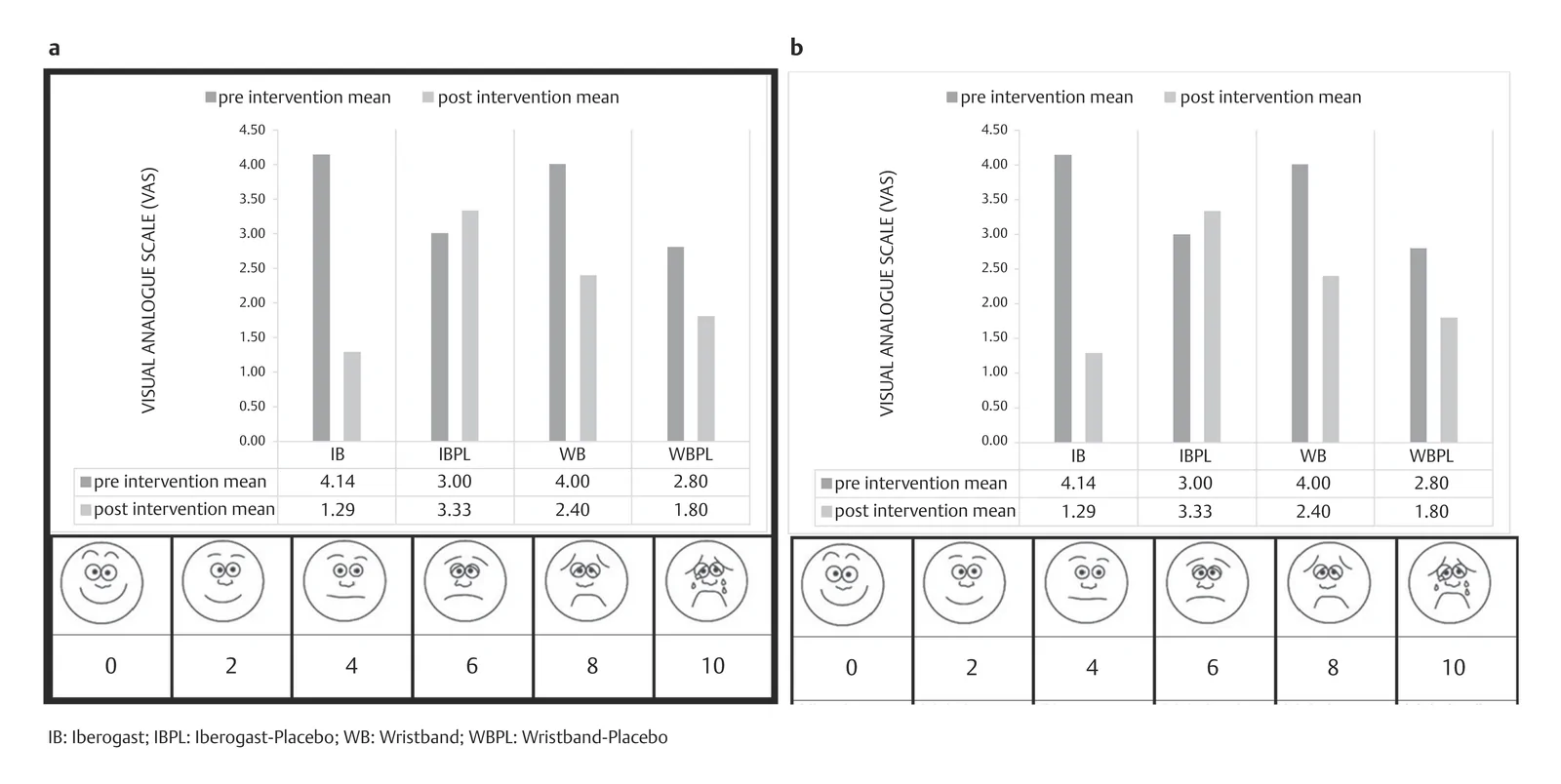
**a** The course of the overall, subjective state of health **b** ) The course of the overall, subjective state of health.

Subjectively, symptoms improved after four weeks of intervention in most treatment-groups: By 68% in children, who received Iberogast (IB), by 40% in the wristband (WB)- group and by 35% in the wrist-band-Placebo-group (WBPL). Patients receiving Iberogast-Placebo (IBPL) showed an increase in symptoms by 10% over the treatment course of four weeks. Fig. 2 illustrates the subjective, overall state of health pre- and postinterventional of the four treatment-groups as described above.

The analysis of the Kidscreen-52 showed, that she sleeping pattern and physical state improved in all four treatment groups, also the number of symptom-free days increased in all patients. Overall happiness showed a positive shift after four weeks of treatment, except for the patients treated with IBPL.

When comparing the results of the Flanker task, there were no statistically significant changes after the intervention, except for the WBPL intervention group, which showed a significant improvement in the accuracy of reaction. All results mentioned and further statistical results can be found in table 2 .

**Table:** **Table 2** Results

			pre-intervention		post-intervention					
			mean (range)	SD	mean (range)	SD	d	F	p	eta2
**Results Questionnaire Quality of life**	**Overall** (degree of symptoms in the previous 7 days, from 0–10 using the VAS)	IB	4.14 (2–6)	2.04	1.29 (0–4)	1.50	1.60			
		IBPL	3.00 (0–6)	2.00	3.33 (0–6)	1.75	− 0.18			
		WB	4.00 (0–8)	1.81	2.40 (0–8)	2.69	0.70			
		WBPL	2.80 (0–6)	1.79	1.80 (0–3)	1.10	0.67			
	**Symptom-free** (on how many days of the previous week was the patient completely without any symptoms; 1–7)	IB	3.14 (0–7)	2.91	4.14 (2–7)	1.68	− 0.42			
		IBPL	2.38 (0–7)	2.88	3.63 (0–7)	2.39	− 0.47			
		WB	2.06 (0–7)	1.92	3.12 (0–7)	2.37	− 0.49			
		WBPL	4.33 (2–7)	1.73	4.78 (2–7)	1.92	− 0.24			
	**Sleep** (difficulty falling/staying asleep, amount of distress about sleep; 0–28 points)	IB	8.86 (4–17)	5.34	6.14 (o-21)	7.27	0.43			
		IBPL	11.25 (2–18)	6.63	9.00 (0–21)	8.23	0.30			
		WB	8.53 (0–19)	5.70	6.16 (0–16)	5.24	0.43			
		WBPL	8.44 (1–20)	5.22	6.44 (0–23)	7.30	0.32			
	**Physical state** (did the patient feel fit and able-bodied, be comfortable; 4–20 points)	IB	15.00 (11–22)	4.58	18.71 (10–22)	4.27	− 0.84			
		IBPL	16.50 (13–23)	4.34	17.25 (12–22)	4.65	− 0.17			
		WB	17.79 (10–24)	4.44	19.11 (7–24)	5.05	− 0.28			
		WBPL	16.67 (12–22)	3.35	18.22 (10–23)	3.99	− 0.42			
	**Emotion** (was the patient happy, content and pleased to be alive in the last 7 days; 6–30 points)	IB	23.00 (19–29)	3.37	25.86 (19–30)	3.63	− 0.82			
		IBPL	25.00 (18–30)	4.21	24.38 (18–29)	4.47	0.14			
		WB	25.32 (15–28)	3.83	26.63 (23–30)	2.54	− 0.40			
		WBPL	23.22 (12–28)	4.94	23.89 (12–30)	6.23	− 0.12			
**Results Flanker-Test**	**Interference** (response time due to inhibition)	IB	0.55	94.94	15.41	63.20	− 0.18	0.18	0.91	0.02
		IBPL	18.99	44.37	31.29	25.69	− 0.34			
		WB	43.43	56.70	52.38	68.01	− 0.14			
		WBPL	− 42.33	164.54	0.43	24.69	− 0.36			
	**Switching** (response time due to switching)	IB	515.54	170.31	589.25	243.64	− 0.35	1.52	0.24	0.16
		IBPL	600.26	112.37	509.99	116.98	0.79			
		WB	598.45	202.72	538.34	125.51	0.36			
		WBPL	471.98	122.96	513.55	111.79	− 0.35			
	**Accuracy** (Accuracy at flanker, corresponds to inhibition)	IB	0.95	0.07	0.99	0.014	0.67	1.16	0.34	0.13
		IBPL	0.95	0.06	0.97	0.01	0.46			
		WB	0.98	0.03	0.95	0.12	− 0.32			
		WBPL	0.83	0.29	0.96	0.04	0.61			
	**Switching accuracy** (Accuracy in switch trials of the flanker task, corresponds to cognitive flexibility)	IB	0.76	0.17	0.86	0.11	0.68	0.96	0.42	0.07
		IBPL	0.89	0.08	0.88	0.08	− 0.12			
		WB	0.90	0.07	0.90	0.11	0.00			
		WBPL	0.90	0.07	0.88	0.09	− 0.30			

One patient complained about a wristband, which was too tight, but he solved the problem by over-stretching it. Otherwise, no adverse events occurred.

## Discussion

This randomized double-blind, placebo-controlled pilot-study in children suffering from FD revealed following key-findings: Over the course of four weeks, patients of all treatment groups had a subjectively improved sleeping pattern, an overall better physical state and were symptom-free on more days per week. All interventions -except for IBPL- also lead to a more positive state of mind and a reduction of symptoms, represented by VAS.

The pathophysiology of FGDI’s is a complex, multifaceted interaction of visceral hypersensitivity, central sensitization, gut microbiota and its metabolic products (such as short-chain fatty acids), early-life programming (surgeries, infections etc.), gastrointestinal motility, neuroimmune interaction 16 and psychological factors.In our study, the objective and subjective findings did not correlate, which is absolutely in line with literature, demonstrating the correlation of psychosocial factors and the presence of FGDI’s: Children and adolescents with FGDI’s tend to have a poorer mental health, elevated levels of anxiety, depression, higher stress levels, exposure to stressful life events, a lower quality of life and present with a higher frequency of extra-intestinal somatic symptoms, such as headaches, fatigue and sleep disturbances, compared to their healthy peers 17 . Also, social contextual factors, such as parental chronic pain, have been associated with a higher frequency of pain episodes in children 18 . Evidence suggests, that parental influence -explained by frameworks such as social learning theory and the social communication model of pain- plays a crucial role in shaping children’s pain perception 19 20 . Meaning, parents may inadvertently encourage their children to mimic their pain expressions, thereby influencing the child’s pain experience and behavior. Given that functional disorders are understood to be multifactorial in origin, with socio-emotional factors and pain management playing a significant role in the perception of pain, it is not surprising that the efficacy of pharmacological approaches (e. g. antispasmodics, antibiotics, antihistaminic, antiemetic) in children with FGDIs is not satisfying 21 .

In this study, most of the patient’s condition subjectively improved regardless of the intervention. One contributing factor might be the security the patients received from learning that the result of the endoscopy was normal, although it was shown that a negative endoscopy alone does not improve the clinical outcome of children with FGIDs 22 . Hollier et al. 23 have shown that somatization and catastrophizing are mediating anxiety and depression, which increases the abdominal discomfort. Therefore, early prevention of somatization by objective parameters could prevent long-term functional abdominal pain, but with the possibility that the tendency to somatization disorder may persist and focus on another organ.

The debate of the role of wristbands in the treatment of nausea is still on-going. A randomized controlled trial of 90 women suffering from hyperemesis gravidarum showed milder symptoms in patients receiving acupressure via wristband vs. the control group having standard medication 24 . A meta-analysis of twelve studies assessing the effectiveness of acupressure in preventing chemotherapy-induced nausea and vomiting was performed 25 : Over 1400 patients were included and showed that acupressure reduced statistically significant the severity of acute and delayed nausea, but no beneficial effect on the incidence or frequency of vomiting was found. A randomized, single-blind, placebo-controlled clinical trial 26 involving 90 patients with acute myocardial infarction and persistent nausea despite the use of anti-emetic medications found a significant (p<0.05) reduction in vomiting in the acupressure group, using the same wrist-bands we used in this study, compared to the placebo and control groups.Lately, acupressure seems to be undergoing an image change to a serious science, which is reflected by a publication in The British Journal of Anaesthesia, the highest-ranking journal worldwide in the field of anaesthesia: Wang et al. 27 performed a study in almost 300 women undergoing hysteroscopic surgery, where besides pain, postoperative nausea and vomiting (PONV) is the most common complication of surgery and anaesthesia. Patients received a bracelet with transcutaneous electrical acupoint stimulation for 24h postoperatively and were compared to patients wearing the same bracelets being switched off. The incidence of vomiting was statistically significant lower in the intervention group (36% vs. 18%, p= 0.003), whereas the nausea was not significantly different.

Whilst adults benefit from anti-nausea wristbands to some extent, literature search revealed that no data from paediatric trials are yet available. In our cohort, children did improve after an intervention with the wristband, however, so did the children wearing WBPL. An extensive education about the disease might explain the good response rate to the WBPL: Not only children, but also parents were intensively informed about the aetiology and the different therapeutical approaches in FD to ensure they do not fear any disadvantages arising from participating in the study. American psychologists 28 tested a cognitive-behavioral intervention delivered to parents of children with FGID’s and found an improvement of the child’s symptoms by educating/strengthening parents. This theory, however, does not explain that the IBPL-group did not benefit from the intervention, although they did receive the same education.

STW-5 (Iberogast), an herbal combination, has been developed over 5 decades ago to treat patients suffering from FD. It was not only shown to increase antral motility 5 , reduce inflammation 29 , but a review of 12 clinical trials of double-blind and randomized studies found statistically significant effects on patient’s symptoms with a comparable efficacy to a standard prokinetic, and a favorable tolerability, which is relevant for long-term treatment 30 . A double-blind, randomized, placebo-controlled crossover trial in adult patients with FD and reflux symptoms demonstrated a significant reduction in the total number of acidic reflux events following four weeks of treatment with STW-5 compared to placebo, confirmed by pH-metry studies 31 . Authors concluded that while STW-5 had no observable effect on oesophageal motility, it significantly reduced acid exposure time. This finding implies that a reduced volume of refluxed acid, possibly due to improved gastric emptying and enhanced gastric motility, may underlie the pharmacological effects of STW-5. This might also explain the benefit our patients experienced, as a swift gastric emptying clearly prohibits fullness and discomfort.

As FD causes considerable strain on many children’s lives, an international pediatric committee performed a systematically assessment of evidence, efficacy and safety of pharmacological treatments of FD in children aged 4–18 years 32 , where no evidence was found to support the use of pharmacological drugs. It was recommended to treat the different subtypes of FD (epigastric pain and postprandial distress syndrome) based on adult data and customized. For the postprandial distress type, the committee recommends the use of fundal relaxant medications, which equals STW-5. Our findings of a good response to STW-5 are therefore not only in line with those recommendation, but also with a prospective observational study of 980 children between 3 and 14 years 33 , where a very good effect of STW-5 was shown with only 0.7% reporting adverse events. STW-5 seems to be a good and safe option for children suffering from FD with a pharmacologic effect, which is underlined by the fact, that the placebo-group did not show any improvement of symptoms, in contrast.

A clear limitation of the present study is the small number of included patients. Although over 200 children were offered participation in the study, only a few agreed to participate in the trial. The most frequently mentioned reasons for declining participation were, that ‘they only wanted to rule out somatic diseases’ and ‘a lack of time’- both reasons were provided by the parents. Also, only a fraction of patients fully completed the 4-week intervention with pre- and post-testing, many participants suddenly did not answer calls and/or Emails and therefore dropped out. From a statistical perspective, this presented us with the problem of an absent statistical significance, indicated as p-values, as this correlates strongly with the sample size. In awareness of this problem, the effect size (eta2) was calculated, which showed a big effect for the overall subjective improvement of condition. The high drop-out rate of our participants might be explained by findings from different studies 34 35 , showing that parents of children with chronic pain struggle with the time consumption of attending doctor and therapy appointments, which is only possible when a strong social support network is in place to provide assistance and flexibility for the changing needs, whilst a low socioeconomic status is associated with a higher frequency of recurrent pain in children 36 37 . In short, the care of children with chronic pain is extremely time-intensive, which can push parents to their limits of capacity and, consequently, leave them feeling overwhelmed by the prospect of participating in a study.

As a side study, participants had to perform the Flanker task. The idea behind this test was our hypothesis, that chronic epigastric discomfort could impair cerebral performance, consequentlyan improvement in symptoms could be objectified with an improved mental function. There are no data available on this subject. Surprisingly, only the WBPL-intervention group, which also showed a subjective improvement in symptoms, showed an improvement in cerebral responsiveness. The data from the Flanker task are not coherent with the reported, subjective gastrointestinal symptoms, which is most likely due to the patient groups being too small.

## Conclusion

The pathophysiology of FD is complex and multifactorial, involving hypersensitivity, altered microbiota and central nervous processing as well as behavioral patterns. Our double-blind, placebo-controlled pilot study showed, that STW-5 and/or anti-nausea wristbands are a cheap and effective option to treat children and teenagers suffering from FD. It certainly is only a method to reduce discomfort selectively, long-term data are not available.

## Contributor’s Statement

C.Légeret and R. Furlano formed the study concept and provided the funding. M. D’Aujourdhui, S. Schneider, A. Acket and H.Köhler performed data acquisition, S. Ludyga performed statistical analysis, C. Légeret wrote the first draft of the manuscript, all authors read and agreed to the latest version of the manuscript.

## Fundref Information

Gottfried and Julia Bangerter-Rhyner-Foundation —

## Notes

Abbreviation **FD** Functional dyspepsia
**FGIDs** Functional gastrointestinal disorders
**IB** Iberogast-Group
**IBPL** IberogastPlacebo-Group
**PONV** postoperative nausea and vomiting
**WB** Wristband-Group
**WBPL** Wristband Placebo-Group
**VAS** Visual Analogue Scale

## References

[1] Vernon-Roberts A Alexander I Day A S Systematic Review of Pediatric Functional Gastrointestinal Disorders (Rome IV Criteria) J Clin Med 2021 10 5087 10.3390/jcm10215087.PMID: 34768604; PMCID: PMC8585107. 34768604 PMC8585107

[2] Schmulson M J Drossman D A What Is New in Rome IV J Neurogastroenterol Motil 2017 23 151 163 10.5056/jnm16214.PMID: 28274109; PMCID: PMC5383110. 28274109 PMC5383110

[3] Ford A C Mahadeva S Talley N J Functional dyspepsia The lancetVolume396 P1689 P1702 2020 10.1016/S0140-6736(20)30469-4 33049222

[4] Vasant D H Whorwell P J Gut-focused hypnotherapy for Functional Gastrointestinal Disorders: Evidence-base, practical aspects, and the Manchester Protocol Neurogastroenterol Motil 2019 31 e13573 10.1111/nmo.13573.Epub 2019 Feb 27. PMID: 30815936; PMCID: PMC6850508 30815936 PMC6850508

[5] Schemann M Landmann M Michel K Effects of the herbal preparation STW 5-II on in vitro muscle activity in the guinea pig stomach Neurogastroenterol Motil 2021 33 e13984 10.1111/nmo.13984 32936513

[6] Schemann M Landmann M Michel K Effects of the herbal preparation STW 5-II on in vitro muscle activity in the guinea pig stomach Neurogastroenterol Motil 2021 33 e13984 10.1111/nmo.13984. 32936513

[7] Radke M Vinson B Lehmann E Functional gastrointestinal disorders in children: Effectivity, safety, and tolerability of the herbal preparation STW-5 (Iberogast ^^®^^ ) in general practice Complementary Therapies in MedicineVolume71 2022 102873ISSN 0965-2299 10.1016/j.ctim.2022.102873 35998755

[8] Thumann T A Pferschy-Wenzig E-M Bauer R Rapid biotransformation of STW 5 constituents by human gut microbiome from IBS- and non-IBS donors Microbiol Spectr 2024 12 e0403123 10.1128/spectrum.04031-23.Epub 2024 May 13. PMID: 38738925; PMCID: PMC11237759 38738925 PMC11237759

[9] Kim Y S Kim J Ryu H S Herbal Therapies in Functional Gastrointestinal Disorders: A Narrative Review and Clinical Implication Front. Psychiatry 2020 11 601 10.3389/fpsyt.2020.00601 32754057 PMC7365888

[10] Raedsch R Vinson B Holtmann G Early onset of efficacy in patients with functional and motility-related gastrointestinal disorders: A noninterventional study with Iberogast ^®^ . Frühzeitiger Wirkungsbeginn bei Patienten mit funktionellen und motilitätsbedingten gastrointestinalen Erkrankungen : Eine nichtinterventionelle Studie mit Iberogast ^®^ Wien Med Wochenschr 2018 168 89 98 10.1007/s10354-017-0578-y 28744774 PMC5820387

[11] Malfertheiner P STW 5 (Iberogast) Therapie in Gastrointestinal functional disorders Dig Dis 2017 35 25 29 10.1159/000485410 29421817

[12] Rowbotham D J Recent advances in the non-pharmacological management of postoperative nausea and vomiting Br J Anaesth 2005 95 77 81 15805141 10.1093/bja/aei125

[13] Parlow J L Meikle A T Avery N Post discharge nausea and vomiting after ambulatory laparoscopy is not reduced by promethazine prophylaxis Can J Anesth 1999 46 719 724 10451129 10.1007/BF03013905

[14] Ravens-Sieberer U Gosch A Kilroe J The KIDSCREEN-52 Quality of Life Measure for Children and Adolescents: Psychometric Results from a Cross-Cultural Survey in 13 European Countries; Value in HealthVolume11 2008 Pages 645 Pages 658 10.1111/j.1524-4733.2007.00291.x 18179669

[15] Ridderinkhof K R Wylie S A van der Molen M W The arrow of time: Advancing insights into action control from the arrow version of the Eriksen flanker task Atten Percept Psychophys 2021 83 700 721 10.3758/s13414-020-02167-z.Epub 2020 Oct 25. PMID: 33099719; PMCID: PMC7884358 33099719 PMC7884358

[16] Cohen J 1988 Statistical Power Analysis for the Behavioral Sciences Hoboken Taylor and Francis

[17] Thapar N Benninga M A Crowell M D Paediatric functional abdominal pain disorders Nat Rev Dis Primers 6 89 2020 10.1038/s41572-020-00222-5 33154368

[18] Newton E Chitkara D K van Tilburg MA L The role of psychological factors in pediatric functional abdominal pain disorders Neurogastroenterol. Motil. 31 e13538 2019 30729663 10.1111/nmo.13538

[19] Sherman A L Bruehl S Walker L S Individual and additive effects of mothers’ and fathers’ chronic pain on health outcomes in young adults with a childhood history of functional abdominal pain J Pediatr Psychol 38 365 375 2013 23335355 10.1093/jpepsy/jss131 PMC3633252

[20] Stone A L Bruehl S Garber J Walker L S Social learning pathways in the relation between parental chronic pain and daily pain severity and functional impairment in adolescents with functional abdominal pain Pain 159 298 305 2018 29016461 10.1097/j.pain.0000000000001085 PMC5889361

[21] Craig K D Social communication model of pain Pain 156 1198 1199 2015 26086113 10.1097/j.pain.0000000000000185

[22] Rexwinkel R de Bruijn CM A Tabbers M M Pharmacologic Treatment in Functional Abdominal Pain Disorders in Children: A Systematic Review Pediatrics 2021 147 e2020042101 10.1542/peds.2020-042101.PMID: 34045320 34045320

[23] Bonilla S Deli Wang Saps M The prognostic value of obtaining a negative endoscopy in children with functional gastrointestinal disorders Clin Pediatr (Phila) 2011 50 396 401 10.1177/0009922810392773Epub 2011 Jan 17. PMID: 21242200 21242200

[24] Hollier J M Multiple psychological factors predict abdominal pain severity in children with irritable bowel syndrome Neurogastroenterol. Motil. 31 e13509 2019 30549152 10.1111/nmo.13509 PMC6651721

[25] Mohd Nafiah N A Nur Azurah A G Effect of Acupressure at P6 on Nausea and Vomiting in Women with Hyperemesis Gravidarum: A Randomized Controlled Trial Int. J. Environ. Res. Public Health 2022 19 10886 10.3390/ijerph191710886 36078602 PMC9518577

[26] Miao J Liu X Wu C Kong H Liu K Effects of acupressure on chemotherapy-induced nausea and vomiting-a systematic review with meta-analyses and trial sequential analysis of randomized controlled trials Int J Nurs Stud 2017 70 27 37 10.1016/j.ijnurstu.2017.02.014.Epub 2017 Feb 14. PMID: 28231440 28231440

[27] Na Wang Peng Ding Yong-Hua Li Wearable transcutaneous electrical acupoint stimulation bracelet for prevention of postoperative nausea and vomiting in patients undergoing hysteroscopic surgery: a randomised controlled trial British Journal of AnaesthesiaVolume129 2022 Pages e85 Pages e87ISSN 0007-0912 10.1016/j.bja.2022.06.028 35933171

[28] Afshar S Khatiban M Hoseini S K The impact of using P6 acupressure on the nausea, vomiting, and comfort of myocardial infarction patients: A randomized, single-blind, placebo-controlled clinical trial Contemp Clin Trials Commun 2023 36 101238 10.1016/j.conctc.2023.101238.PMID: 38144876; PMCID: PMC10746401. 38144876 PMC10746401

[29] Levy R L Langer S L Feld A D Brief telephone-delivered cognitive behavioral therapy targeted to parents of children with functional abdominal pain: a randomized controlled trial Pain 2017 158 618 628 10.1097/j.pain.0000000000000800.PMID: 28301859; PMCID: PMC5370191 28301859 PMC5370191

[30] Ottillinger B Storr M Allescher H D STW 5 (Iberogast ^®^ ) – a safe and effective standard in the treatment of functional gastrointestinal disorders Wien Med Wochenschr 2013 163 65 72 10.1007/s10354-012-0169-x.Epub 2012 Dec 20PMID: 23263639; PMCID: PMC3580135 23263639 PMC3580135

[31] Mohamed S S Abdeltawab N F Wadie W Effect of the standard herbal preparation, STW5, treatment on dysbiosis induced by dextran sodium sulfate in experimental colitis BMC Complement Med Ther 21 168 2021 10.1186/s12906-021-03337-8 34103031 PMC8188707

[32] Browne P D Nagelkerke SC J Tabbers M M Pharmacological treatments for functional nausea and functional dyspepsia in children: a systematic review Expert Rev Clin Pharmacol 2018 11 1195 1208 10.1080/17512433.2018.1540298.Epub 2018 Dec 6. PMID: 30360666 30360666

[33] Oude Nijhuis RA B Kuipers T Bredenoord A J The Effect of STW5 (Iberogast) on Reflux Symptoms in Patients With Concurrent Dyspeptic Symptoms: A Double-blind Randomized Placebo-controlled Crossover Trial J Neurogastroenterol Motil 2024 30 54 63 10.5056/jnm23014.Epub 2023 Dec 2. PMID: 38043927; PMCID: PMC10774799. 38043927 PMC10774799

[34] Radke M Vinson B Lehmann E Functional gastrointestinal disorders in children: Effectivity, safety, and tolerability of the herbal preparation STW-5 (Iberogast ^^®^^ ) in general practice Complementary Therapies in MedicineVolume71 2022 102873ISSN 0965-2299 35998755 10.1016/j.ctim.2022.102873

[35] Le A Dick B R Spiers J Scott S D Parents’ experiences with pediatric chronic pain Can J Pain 2019 3 20 32 10.1080/24740527.2019.1577679PMID: 35005391; PMCID: PMC8730635 35005391 PMC8730635

[36] Skarstein S Bergem A K Helseth S How do mothers of adolescents with chronic pain experience their own quality of life? BMC Psychol 8 64 2020 10.1186/s40359-020-00430-4 32546203 PMC7298795

[37] Petri L Poulain T Vogel M Hiemisch A Parent-perceived recurrent pain in children: associations with maternal pain, depressiveness, socioeconomic status, and children’s behavioural difficulties Front Pediatr 2024 12 1.287343E6 10.3389/fped.2024.1287343.PMID: 38379914; PMCID: PMC10876899. PMC10876899 38379914

